# Effects of tick surveillance education on knowledge, attitudes, and practices of local health department employees

**DOI:** 10.1186/s12889-022-12667-2

**Published:** 2022-02-02

**Authors:** Lee Ann Lyons, Nohra Mateus-Pinilla, Rebecca L. Smith

**Affiliations:** 1 Department of Pathobiology, University of Illinois, 2001 S. Lincoln Avenue, Urbana, IL 61802 USA; 2 University of Illinois, Illinois Natural History Survey-Prairie Research Institute, 1816 S. Oak Street, Champaign, IL 61820 USA; 3 University of Illinois, Carle-Illinois College of Medicine, 807 S Wright St, Champaign, IL 61820 USA

**Keywords:** Tick surveillance, Tick-borne disease surveillance, Training, Local public health, Knowledge attitudes practices (KAP) survey

## Abstract

**Background:**

The number of cases of tick-borne diseases in humans is increasing rapidly within Illinois. The responsibility for increased surveillance of tick-borne disease cases and tick vectors is being placed on local health departments throughout the United States, but they often lack the funding, time, and/or training needed to perform said surveillance. The aims of this study were to develop, deliver, and determine the effectiveness of tick surveillance training workshops for local health department employees within Illinois.

**Methods:**

We developed and delivered in-person training at local health department offices in each of six Illinois Department of Public Health Environmental Health Regions between April–May of 2019. Pre-, post-, and six-month follow-up questionnaires on knowledge, attitudes, and practices with regards to tick surveillance were administered to training participants. Paired student’s t-test or Wilcoxon signed-rank test were used to compare knowledge, attitudes, and practices scores between questionnaires with Cohen’s d being used to calculate effect sizes associated with t-tests. McNemar’s and McNemar-Bowker tests were used to evaluate individual questions. Spearman’s rank correlation was used to evaluate the relationship between knowledge, attitudes, and practices at pre-, post-, and six-month follow-up.

**Results:**

Seventy-six employees from 40 local health departments that represent 44% (45/102) of Illinois counties attended at least one training workshop. Of these attendees, 81.5% (62/76) participated in at least one survey, 79% (60/76) in the in-person pre-training survey, 74% (56/76) in the in-person post-training survey, and 22% (17/76) in the online six-month follow-up survey. The average knowledge score was significantly increased by 8.21 (95% CI:7.28–9.14) points from pre-training to post-training. The average overall attitude score significantly increased by 5.29 (95% CI: 3.91–6.66) points from pre- to post-training. There were no significant differences in practice scores.

**Conclusions:**

Our study found the training was effective in increasing the knowledge of ticks, tick-borne diseases, and surveillance as well as promoting positive attitudes related to surveillance. While the training, by itself, was not associated with increases in surveillance practices, we were able to empower local public health officials with the knowledge and positive attitudes needed to enact change.

**Supplementary Information:**

The online version contains supplementary material available at 10.1186/s12889-022-12667-2.

## Background

Cases of tick-borne disease (TBDs) such as Lyme disease, spotted fever group rickettsiosis, ehrlichiosis, and anaplasmosis have been increasing throughout Illinois since the 1990s [1–4]. These increases in TBDs can be partially attributed to the geographic expansion of vector tick species within Illinois [5–10]. Experiencing a tick encounter is associated with increased risk of infection with a TBD [11, 12]. The vector species of most concern in Illinois are the hard-bodied (Ixodida: Ixodidae) ticks *Amblyomma americanum* (L.) (Ixodida: Ixodidae), *Amblyomma maculatum* (Koch) (Ixodida: Ixodidae), *Dermacentor variabilis* (Say) (Ixodida: Ixodidae), and *Ixodes scapularis* (Say) (Ixodida: Ixodidae).

Determining when and where people are being exposed to ticks as well as what pathogens those ticks are transmitting has become key in the diagnosis and prevention of TBDs. Preventing tick bites by avoiding high-risk habitats during peak tick activity periods and using personal protective measures when exposure to tick habitats cannot be avoided are the main strategies for prevention and control [13]. Tick surveillance is therefore an essential tool for public health. Surveillance provides information on tick and tick-borne pathogen distributions, can be used to predict trends and risks of exposure to ticks and TBDs, and facilitates monitoring for newly emerging or re-emerging pathogens [14].

Tick surveillance has historically lacked systematic sampling guidelines leading to the loss of data or to the acquisition of data lacking in enough details (e.g. precise geographic location, life stage, quantity of ticks, collection date) to provide complete and/or reliable distribution maps of ticks and tick-borne pathogen [15]. A survey of vector-borne disease professionals in the US, carried out by the Centers for Disease Control and Prevention (CDC)-funded Centers of Excellence in Vector Borne Diseases, found that less than 50% of respondents conducted active tick surveillance [16]. Some of the main barriers to surveillance for local or county level public health agencies included lack of funding, lack of trained personnel, and/or lack of guidelines for best practices. Within months of the completion of that survey, the CDC published guidelines for surveillance of *Ixodes scapularis* or *I. pacificus* and their associated pathogens, followed a year later by guidelines for metastriate (non-*Ixodes* hard tick genera) ticks [17, 18]. The CDC guidelines recommend that tick and TBD surveillance efforts should include allocating funding and providing training to local public health agencies [14, 16].

Recognizing the need for increased active tick surveillance in Illinois and the lack of trained personnel at the local level, the objectives of this work were to: 1) develop a training for local health departments of tick surveillance; 2) deliver in-person training to local health department (LHD) employees; 3) administer a Knowledge, Attitudes, and Practices (KAP) survey before and after the training; and 4) test the hypothesis that providing in-person educational training on tick surveillance will improve knowledge and attitudes of local health department personnel, leading to increases in active tick surveillance within their jurisdiction.

## Methods

### Study design and settings

In cooperation with the Illinois Department of Public Health (IDPH), we developed and delivered in-person training for LHD employees throughout the state. Workshops were held at LHD offices in each of six IDPH Environmental Health Regions between April–May of 2019. These locations were based on the timing of the workshops and the logistics associated with holding meetings in government building (i.e., access, meeting room availability, employee availability). Participants for the training workshops were recruited through email correspondence and flyers provided by IDPH to all the LHDs in the state. Participation in the training workshops was voluntary. Each workshop was three hours long and included five modules covering tick ecology and tick identification, tick-borne diseases in Illinois, tick surveillance methods, tick surveillance safety, and reporting tick surveillance data. Training materials were developed based on the CDC guidelines, “Surveillance for *Ixodes scapularis* and pathogens found in this tick species in the United States”, published in 2019 [17]. Educational materials included the use of images, video examples, and practical experience with tick identification. Microscopes as well as physical specimens of mobile life stages of the four main vector species in Illinois (*A. americanum*, *A. maculatum*, *D. variabilis*, *I. scapularis*) were provided for tick identification practice, although the only larval specimen was *A. americanum* and only adult *A. maculatum* were available.

Prior to the start of every workshop, attendees were asked if they would voluntarily participate in a study on knowledge, attitudes, and practices (KAP) of tick surveillance and written informed consent was obtained from each participant. The University of Illinois Institutional Review Board reviewed the protocol for the study and found it to be exempt from formal review (IRB #19,625). Questionnaires were administered to participants pre-workshop, immediately post-workshop, and at least six months after the workshops. The pre- and post- questionnaires were paper-based and provided on-site. In November 2019, participants were sent a link to the last questionnaire, administered through Google Forms, via email.

### Study instruments

The pre- and post- questionnaires consisted of 42 questions (*n* = 2 demographic, *n* = 26 knowledge-based, *n* = 12 attitude-based, and *n* = 2 practice-based). All the knowledge-based and one of the practice-based questions were multiple choice or true/false. All the attitude-based and one of the practice-based questions were Likert scale questions scaled from strongly agree to strongly disagree. The six-month follow-up questionnaire was the same as the previous two questionnaires except for the last question, which asked what actual surveillance was performed in 2019 as opposed to what surveillance was planned. Knowledge questions covered tick ecology and identification (*n* = 7), tick surveillance methods (*n* = 6), safety when performing tick surveillance (*n* = 6), and tick-borne diseases found in Illinois (*n* = 7). Attitude questions dealt with perceived need and barriers to performing tick surveillance as well as the perceived risk of tick-borne diseases in the state and participant’s jurisdiction. Cronbach’s alpha analysis was used to confirm internal reliability of the questions assessing attitudes. The perceived needs subscale consisted of 4 items (*α* = 0.73), the perceived barriers subscale consisted of 5 items (*α* = 0.75), and the perceived risk of TBD subscale consisted of 3 items (*α* = 0.80). Practice-based questions covered what surveillance was planned and/or has been performed and interest in increasing surveillance (*n* = 2).

### Statistical analysis

Descriptive statistics were calculated to provide a summary of responses. Knowledge questions were given a score of one for a correct response and a score of zero for a wrong or unanswered response for a total of 26 points. The questions were then divided into subgroups (i.e., ticks and tick identification, tick surveillance methods, tick surveillance safety, and tick-borne disease) and subtotals were calculated. Likert questions were scored by participants as strongly agree, agree, neutral, disagree, or strongly disagree. Statements with positive attitudes or practices towards surveillance had strongly agree scored as (5), agree as (4), neutral (3), disagree (2), and strongly disagree (1). The scores of statements with negative attitudes were scored in the reverse with a total possible score of 60 points. Attitude questions were also subdivided into categories (i.e., need for surveillance, barriers towards performing surveillance, risk of tick-borne diseases in jurisdiction) for further analysis. The second practice question was scored (2) for “Yes” performing or planning to perform tick surveillance, (1) for “Maybe” planning to perform surveillance, and (0) for “No” performing or planning to perform surveillance or no response making a total possible practices (P) score of seven. Category scores for knowledge (K) and attitudes (A) were calculated for each participant by totaling scores from questions associated with each one. Paired student’s t-test (normally distributed) or Wilcoxon signed-rank test (non-normally distributed) were used to compare total K, A, and P scores, and K and A category scores. Cohen’s d was used to calculate effect sizes associated with t-tests. McNemar’s and McNemar-Bowker tests were used to evaluate individual K, A, and P questions. A *p*-value of < 0.05 on two-tailed testing was considered statistically significant. Spearman’s rank correlation was used to evaluate the relationship between knowledge, attitudes, and practices at pre- and post-training. Statistical analysis was performed using RStudio version 1.2.1335 [19].

## Results

**Fig. 1:**
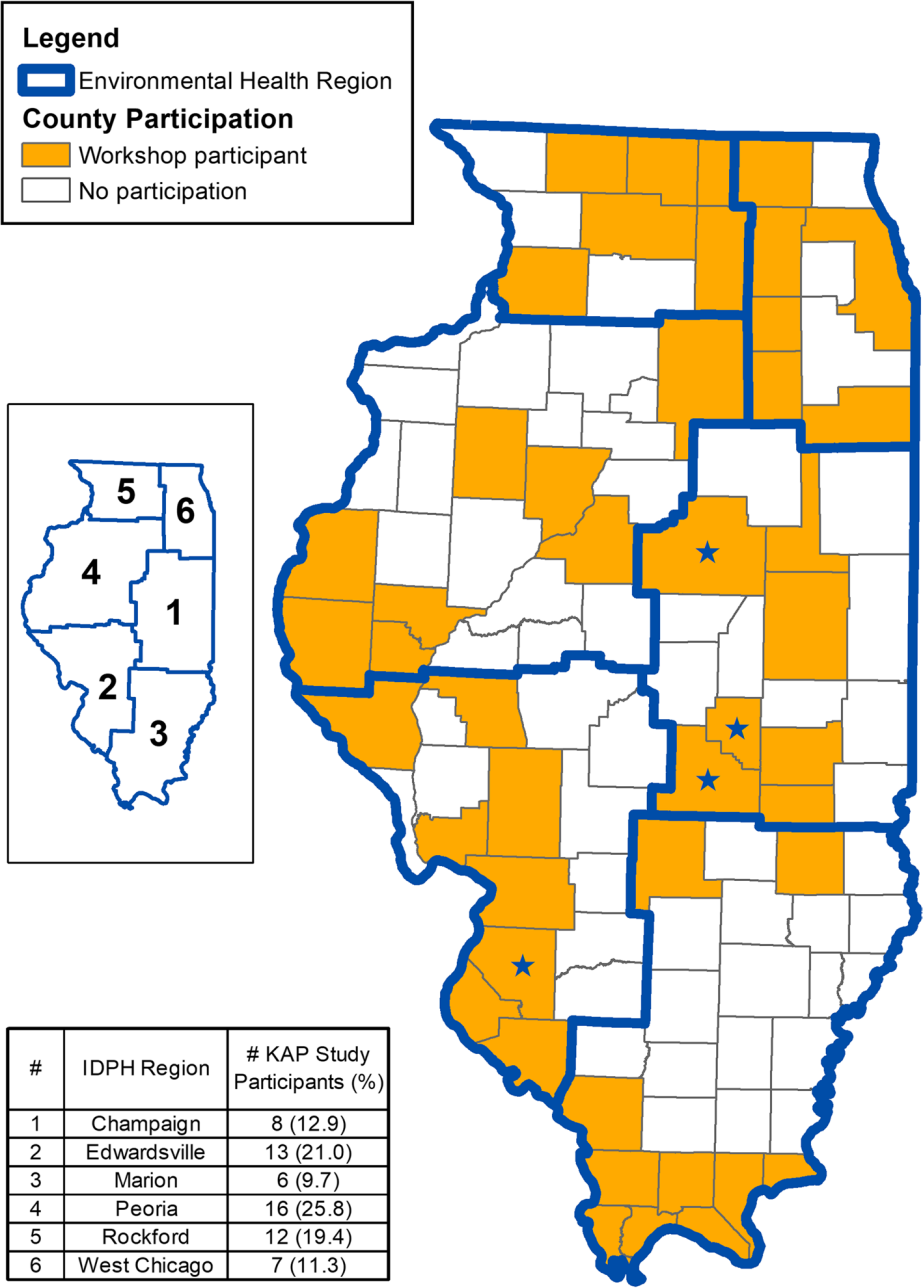
Counties Represented by Workshop Participants*.* Counties with a blue star had participants that did the training but did not participate in the KAP study

Seventy-six employees from 40 different local health departments that represent 44% (45/102) of Illinois counties, attended at least one of the six training workshops held between April–May 2019 (Fig. 1). Of these attendees, 81.5% (62/76) participated in at least one survey, 79% (60/76) in the in-person pre-training survey, 74% (56/76) in the in-person post-training survey, and 22% (17/76) in the online six-month follow-up survey. There was at least one participant in the study from every IDPH Environmental Health Region, but the highest number of participants were from the Peoria region (25.8%, 16/62) followed by the Edwardsville region (21.0%, 13/62) (Fig. 1). Most of the participants worked in the Environmental Health Division (85.5%, 53/62) of their LHD (Table 1). Respondents that completed both the pre- and post- surveys or completed all three surveys made up 67.7% (42/62) and 19.4% (12/62) of study participants, respectively.

**Table 1:** Study Participants Employment Demographics

	Pre-training (*N*=60)	Post-training (*N*=56)	Six-months follow-up (*N*=17)
Response	Number (%)	Number (%)	Number (%)
**IDPH Environmental Health Region of Participant**			
Champaign	8 (13.3)	7 (12.5)	0 (0.0)
Edwardsville	11 (18.3)	10 (17.9)	4 (23.5)
Marion	6 (10.0)	6 (10.7)	2 (11.8)
Peoria	16 (26.7)	15 (26.8)	7 (41.2)
Rockford	12 (20.0)	11 (19.6)	3 (17.6)
West Chicago	7 (11.7)	7 (12.5)	1 (5.9)
**Health Department**			
Adams County	1 (1.7)	1 (1.8)	2 (11.8)
Brown County	1 (1.7)	1 (1.8)	0 (0.0)
Boone County	1 (1.7)	1 (1.8)	0 (0.0)
Champaign-Urbana Public Health District	4 (6.7)	3 (5.4)	0 (0.0)
Coles County	1 (1.7)	1 (1.8)	0 (0.0)
Cumberland County	1 (1.7)	1 (1.8)	0 (0.0)
Dekalb County	1 (1.7)	1 (1.8)	0 (0.0)
Fayette County	1 (1.7)	1 (1.8)	0 (0.0)
Ford County	2 (3.3)	2 (3.6)	0 (0.0)
Grundy County	2 (3.3)	2 (3.6)	0 (0.0)
Hancock County	1 (1.7)	1 (1.8)	1 (5.9)
Jackson County	3 (5.0)	3 (5.4)	1 (5.9)
Jasper County	1 (1.7)	1 (1.8)	1 (5.9)
Jersey County	1 (1.7)	1 (1.8)	0 (0.0)
Kane County	1 (1.7)	1 (1.8)	0 (0.0)
Kankakee County	2 (3.3)	1 (1.8)	0 (0.0)
Kendall County	2 (3.3)	1 (1.8)	2 (11.8)
Knox County	2 (3.3)	2 (3.6)	0 (0.0)
LaSalle County	3 (5.0)	2 (3.6)	1 (5.9)
Macoupin County	1 (1.7)	1 (1.8)	0 (0.0)
Madison County	3 (5.0)	3 (5.4)	1 (5.9)
McHenry County	0 (0.0)	1 (1.8)	0 (0.0)
Monroe County	1 (1.7)	1 (1.8)	1 (5.9)
Morgan County	1 (1.7)	1 (1.8)	0 (0.0)
Ogle County	2 (3.3)	2 (3.6)	0 (0.0)
Peoria City/County	2 (3.3)	2 (3.6)	0 (0.0)
Pike County	2 (3.3)	1 (1.8)	1 (5.9)
Randolph County	1 (1.7)	1 (1.8)	1 (5.9)
Schuyler County	1 (1.7)	1 (1.8)	0 (0.0)
Southern Seven	1 (1.7)	1 (1.8)	0 (0.0)
Stephenson County	2 (3.3)	2 (3.6)	2 (11.8)
Stickney Township	1 (1.7)	1 (1.8)	0 (0.0)
Tazewell County	5 (8.3)	5 (8.9)	2 (11.8)
Whiteside County	1 (1.7)	1 (1.8)	0 (0.0)
Winnebago County	5 (8.3)	5 (8.9)	1 (5.9)
**Health Department Division**			
Environmental Health	51 (85.0)	48 (85.7)	15 (88.2)
Community Health	5 (8.3)	5 (8.9)	2 (11.8)
Emergency Response	2 (3.3)	1 (1.8)	0 (0.0)
Administration	2 (3.3)	2 (3.6)	0 (0.0)

### Knowledge

Of all the participants in the KAP study, only one respondent (1.8%) correctly answered all the knowledge questions in the post-training questionnaire (*n* = 56), but there were zero in the pre-training (*n* = 60) and follow-up questionnaires (*n* = 17). No respondents who completed both the pre- and post- (*n* = 42) or completed all three questionnaires (*n* = 12) answered all the knowledge questions correctly. The only question that all respondents got correct in the post- (56/56) and follow-up (17/17) questionnaires was “The "fingers" or "strips" on a drag along with weights sewn into the trailing edge are there to increase contact between the fabric and vegetation”. All the follow-up respondents also correctly identified the picture of a lone star tick. The only other question with > 90% correct responses in all three questionnaires was “There are many other safety concerns involved with tick surveillance besides tick bites” of which there were only four participants who answered incorrectly during the pre-questionnaire. The question with the most incorrect responses pre-training (88.3%) and post-training (92.9%) was “Which of the following are true about tick-borne diseases?”. Six-months after training, the question with the most incorrect responses (88.2%) was “Which of the following are acceptable tick collection methods for classifying the county status of tick species?” (Suppl Table 1).

**Fig. 2:**
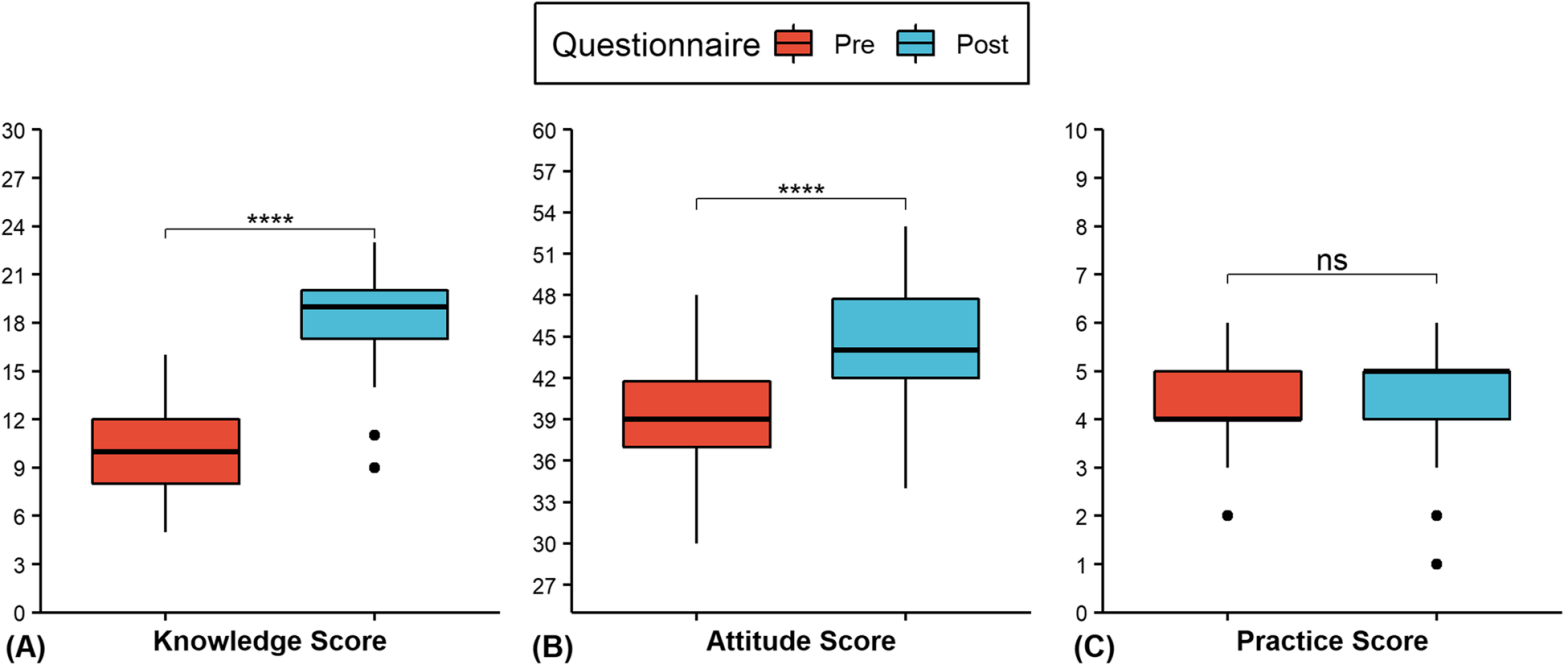
Distribution of knowledge (**A**), attitudes (**B**), and practices (**C**) scores for respondents who completed both pre and post questionnaires (black dots represent outlier scores). Significance level from Paired Student t-test for knowledge and attitudes scores and Wilcoxon signed rank test for practice scores: ****p < 0.0001

**Fig. 3:**
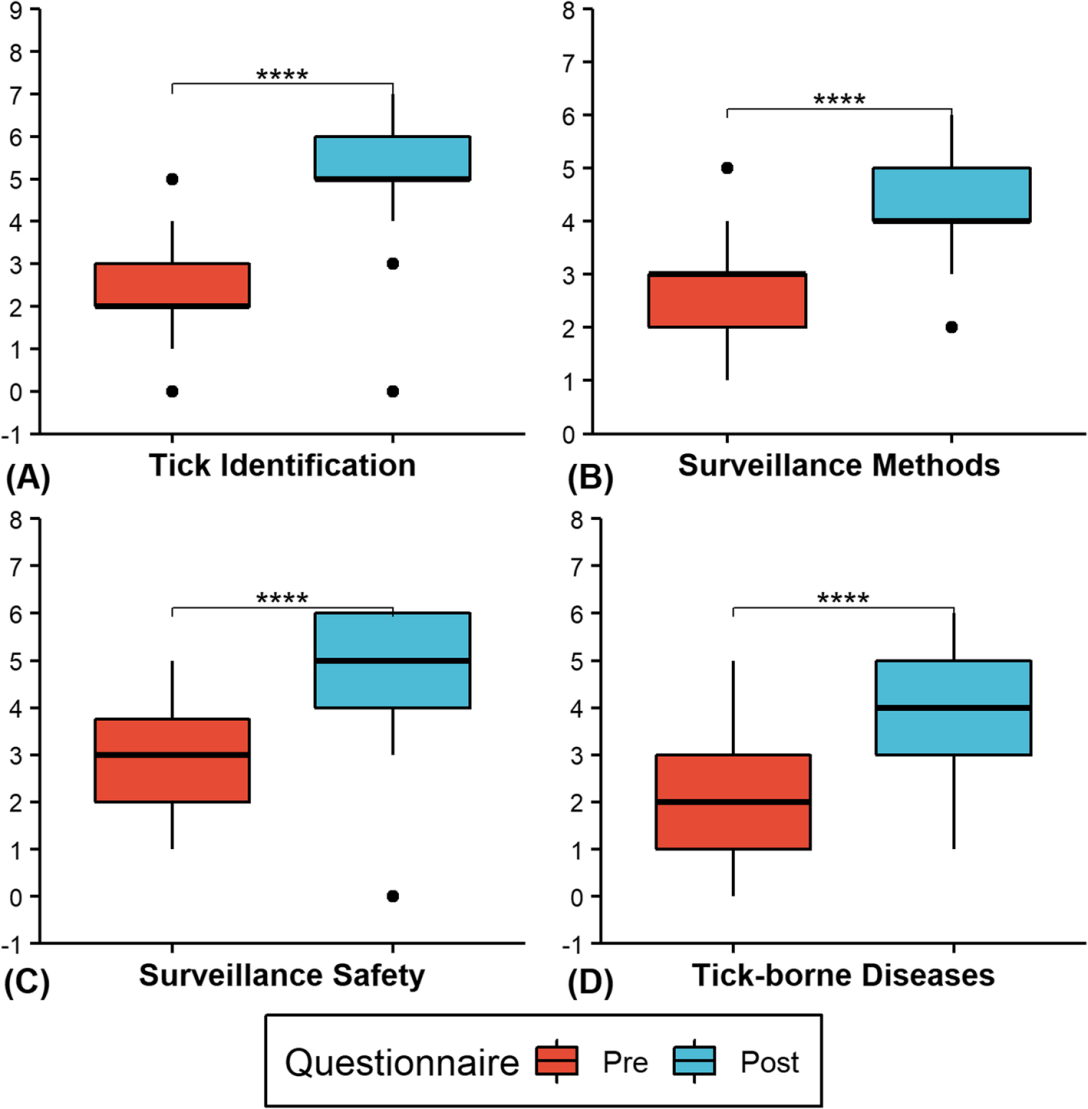
Distribution of knowledge scores from pre- to post-training by knowledge subcategories: (**A**) tick identification, (**B**) surveillance methods, (**C**) surveillance safety, (**D**) tick-borne diseases (black dots represent outlier scores). Significance level from Paired Student t-test: ****p < 0.0001

Zero respondents who completed both the pre- and post-surveys (*N* = 42) answered greater than 70% of the knowledge questions correctly pre-training, but 52.4% (22/42) did after training. The proportion of participants who changed from the incorrect to the correct response pre- to post-training was statistically significant (*p* < 0.05) for 17 (65.4%) of the knowledge questions (Table 2). Additionally, the average knowledge score was significantly (t (41) = 17.84, *p* < 0.0001, d = 2.75) increased by 8.21 (95% CI:7.28–9.14) points from pre-training (M = 10.00, SD = 2.61) to post-training (M = 18.21, SD = 2.80) (Fig. 2). Significant (*p* < 0.0001) increases in mean knowledge scores occurred in all subcategories immediately following training (Fig. 3 & Table 3).

**Table 2:** Paired knowledge responses to pre- and post- questionnaires

		**Pre-training(*N* = 42)**	**Post-training(*N* = 42)**	**Pre vs Post**
**Question**	**Response**	**Number (%)**	**Number (%)**	***P*-value**
**Which tick life stages have 8 legs? (Select all that apply)**	Incorrect	31 (73.8)	8 (19.0)	< 0.0001
	Correct	11 (26.2)	34 (81.0)	
**The "head" of a tick is called the hypostome and is made up of the palps, the chelicerae, and the capitulum. (True/False)**	Incorrect	35 (83.3)	31 (73.8)	0.4227
	Correct	7 (16.7)	11 (26.2)	
**What sex is the tick in the picture below? (Male/Female)**	Incorrect	16 (38.1)	1 (2.4)	0.0003
	Correct	26 (61.9)	41 (97.6)	
**What tick species is pictured below? (Select species)**	Incorrect	17 (40.5)	7 (16.7)	0.0044
	Correct	25 (59.5)	35 (83.3)	
**Which species of tick has an anal groove seen in the picture below? (Select species)**	Incorrect	36 (85.7)	12 (28.6)	< 0.0001
	Correct	6 (14.3)	30 (71.4)	
**What are the ridges called that run along the bottom abdominal border of some tick species (see picture below)? (Select from the following)**	Incorrect	35 (83.3)	1 (2.4)	< 0.0001
	Correct	7 (16.7)	41 (97.6)	
**Which of the following is an insecticide? (Select one)**	Incorrect	22 (52.4)	4 (9.5)	< 0.0001
	Correct	20 (47.6)	38 (90.5)	
**All of the following are false when removing an attached tick except? (Select one)**	Incorrect	30 (71.4)	18 (42.9)	0.0033
	Correct	12 (28.6)	24 (57.1)	
**Which of the following are common places to find ticks on your body? (Select all that apply)**	Incorrect	26 (61.9)	5 (11.9)	< 0.0001
	Correct	16 (38.1)	37 (88.1)	
**After treating clothing with permethrin, you must allow it to dry for at least 12 h before wearing. (True/False)**	Incorrect	27 (64.3)	11 (26.2)	0.0008
	Correct	15 (35.7)	31 (73.8)	
**There are many other safety concerns involved with tick surveillance besides tick bites. (True/False)**	Incorrect	4 (9.5)	2 (4.8)	0.6831
	Correct	38 (90.5)	40 (95.2)	
**If performing tick surveillance during hunting season, which of the following is false? (Select one)**	Incorrect	28 (66.7)	9 (21.4)	< 0.0001
	Correct	14 (33.3)	33 (78.6)	
**The four most common tick-borne disease in Illinois are Lyme disease, Anaplasmosis, Tularemia, and Ehrlichiosis (True/False)**	Incorrect	32 (76.2)	29 (69.0)	0.5465
	Correct	10 (23.8)	13 (31.0)	
**Which of the following are true about tick-borne diseases? (Select all that apply)**	Incorrect	38 (90.5)	40 (95.2)	0.4795
	Correct	4 (9.5)	2 (4.8)	
**Which of the following is not a tick-borne disease? (Select one)**	Incorrect	32 (76.2)	4 (9.5)	< 0.0001
	Correct	10 (23.8)	38 (90.5)	
**Erythema migrans ("bullseye" rash) is only seen with Lyme disease (see picture below). (True/False)**	Incorrect	28 (66.7)	15 (35.7)	0.0009
	Correct	14 (33.3)	27 (64.3)	
**Which pathogen causes Rocky Mountain Spotted Fever? (Select one)**	Incorrect	15 (35.7)	7 (16.7)	0.0613
	Correct	27 (64.3)	35 (83.3)	
**Within Illinois, which tick species is most likely to transmit Anaplasmosis? (Select species)**	Incorrect	31 (73.8)	18 (42.9)	0.0088
	Correct	11 (26.2)	24 (57.1)	
**How many hosts do the vector ticks of concern in Illinois require to complete their lifecycle? (Select one)**	Incorrect	33 (78.6)	13 (31.0)	< 0.0001
	Correct	9 (21.4)	29 (69.0)	
**Which tick species will you encounter more frequently in grassy fields? (Select species)**	Incorrect	23 (54.8)	16 (38.1)	0.2109
	Correct	19 (45.2)	26 (61.9)	
**When is a tick species considered to be established within a county? (Select one)**	Incorrect	26 (61.9)	12 (28.6)	0.0080
	Correct	16 (38.1)	30 (71.4)	
**Which of the following are acceptable tick collection methods for classifying the county status of tick species? (Select all that apply)**	Incorrect	34 (81.0)	13 (31.0)	< 0.0001
	Correct	8 (19.0)	29 (69.0)	
**How often should drags be inspected for ticks (according to CDC guidelines) when you want to calculate the density of host-seeking nymphs? (Select one)**	Incorrect	26 (61.9)	20 (47.6)	0.3074
	Correct	16 (38.1)	22 (52.4)	
**How many sites must be sampled per county (according to CDC guidelines) when you want to calculate density of host-seeking females? (Select one)**	Incorrect	37 (88.1)	28 (66.7)	0.0159
	Correct	5 (11.9)	14 (33.3)	
**Ticks can be preserved in 70–95% ethanol. (True/False)**	Incorrect	6 (14.3)	3 (7.1)	0.4497
	Correct	36 (85.7)	39 (92.9)	
**The "fingers" or "strips" on a drag along with weights sewn into the trailing edge are there to increase contact between the fabric and vegetation. (True/False)**	Incorrect	4 (9.5)	0 (0.0)	0.1336
	Correct	38 (90.5)	42 (100.0)	
**Knowledge Score (Mean (SD))**		10.00 (2.61)	18.21 (2.80)	< 0.0001

**Table 3:** Mean and median scores for knowledge and attitude subcategories of participants who completed both pre- and post- questionnaires

	**Pre-training (*N* = 42)**	**Post-training (*N* = 42)**
Knowledge	**Mean (SD)**	**Mean (SD)**
Ticks and tick identification (*n* = 7 questions)	2.40 (1.13)	5.19 (1.19)
Surveillance methods (*n* = 6 questions)	2.83 (0.91)	4.19 (0.92)
Surveillance safety (*n* = 6 questions)	2.73 (1.21)	4.83 (1.23)
Tick-borne diseases (*n* = 7 questions)	2.02 (1.22)	4.00 (1.27)
Attitudes	**Median (IQR)**	**Median (IQR)**
Perceived need (*n* = 4 questions)	16 (14.25–15.45)	16 (15–17)
Perceived barriers (*n* = 5 questions)	11 (10–13.75)	16 (14–18)
Perceived risk (*n* = 3 questions)	12 (11.25–13)	12 (12–14)

*SD* Standard deviation, *IQR* Interquartile range

### Attitudes

Sixty-five percent or more of all pre- (39/60), post- (41/56), and follow-up (11/17) participants were worried about tick-borne diseases within Illinois, and believed TBDs were a problem within their jurisdiction (Suppl Table 2). Sixty percent of participants also believed their department was not currently doing enough tick surveillance even though $\ge$ 75% at all three timepoints agreed it was needed within their jurisdiction. Lack of time was the barrier with the highest proportions of agreement across time (40% pre, 46% post, 59% follow-up).

**Fig. 4:**
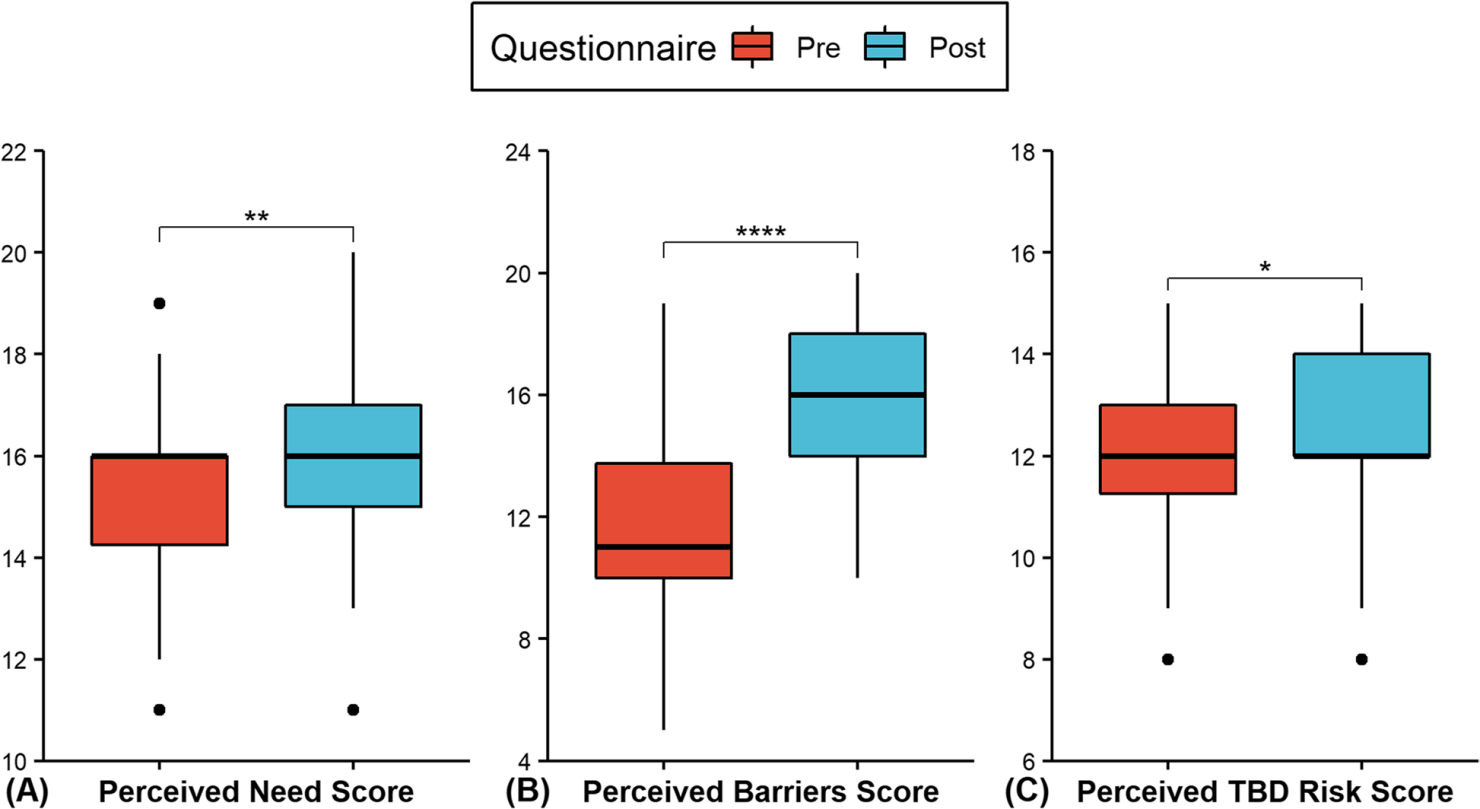
Distribution of attitude scores grouped by subcategories: (**A**) perceived need, (**B**) perceived barriers, (**C**) perceived TBD risk (black dots represent outlier scores). Significance level from Wilcoxon signed-rank test: * *p* < 0.05, ** *p* < 0.01, *** *p* < 0.001, ****p < 0.0001

The average overall attitude score significantly (t (41) = 7.75, *p* < 0.0001, d = 1.20) increased by 5.29 (95% CI: 3.91–6.66) points from pre- (M = 39.10, SD = 4.11) to post-training (M = 44.38, SD = 4.37) (Fig. 2). Significantly higher medians pre- to post-training were also observed when the questions were grouped into perceived need for surveillance (Z = 108, *p* = 0.0095), barriers to performing surveillance (Z = 30, *p* < 0.0001), and risk of TBD (Z = 31, *p* = 0.0153) (Fig. 4 & Table 3). Only one question, “I feel confident I can identify the four main vector tick species within IL.”, had a significant (*p* = 0.0398) pairwise comparison with respondents moving from disagree prior to training to agree afterward (Table 4).

**Table 4:** Paired responses to attitudes questions for pre- and post- questionnaires

		**Pre-training** **(*N* = 42)**	**Post-training** **(*N* = 42)**	
**Question**	**Response**	**Number (%)**	**Number (%)**	**P-value**
**Tick surveillance is needed within my jurisdiction**	Strongly Disagree	0 (0.0)	0 (0.0)	0.1841 N to A A to StA
	Disagree	1 (2.4)	1 (2.4)	
	Neutral	7 (16.7)	2 (4.8)	
	Agree	31 (73.8)	30 (71.4)	
	Strongly Agree	3 (7.1)	9 (21.4)	
**My department is already doing enough tick surveillance**	Strongly Agree	0 (0.0)	1 (2.4)	0.3428 N to D
	Agree	3 (7.1)	3 (7.1)	
	Neutral	12 (28.6)	8 (19.0)	
	Disagree	21 (50.0)	25 (59.5)	
	Strongly Disagree	6 (14.3)	5 (11.9)	
**I do not feel like I have enough knowledge and preparation to do tick surveillance accurately**	Strongly Agree	12 (28.6)	1 (2.4)	0.0933 A too D 0.0940 A too N
	Agree	22 (52.4)	7 (16.7)	
	Neutral	3 (7.1)	12 (28.6)	
	Disagree	5 (11.9)	21 (50.0)	
	Strongly Disagree	0 (0.0)	1 (2.4)	
**I do not feel like I have enough knowledge and preparation to do tick surveillance safely**	Strongly Agree	11 (26.2)	0 (0.0)	0.0934 StA to D A too D
	Agree	14 (33.3)	5 (11.9)	
	Neutral	10 (23.8)	9 (21.4)	
	Disagree	7 (16.7)	26 (61.9)	
	Strongly Disagree	0 (0.0)	2 (4.8)	
**I feel confident I can identify the four main vector tick species within IL**	Strongly Disagree	11 (26.2)	0 (0.0)	0.0398 D to A
	Disagree	18 (42.9)	4 (9.5)	
	Neutral	4 (9.5)	11 (26.2)	
	Agree	7 (16.7)	25 (59.5)	
	Strongly Agree	2 (4.8)	2 (4.8)	
**I do not feel like I have enough time for tick surveillance within my job**	Strongly Agree	7 (16.7)	6 (14.3)	0.0700 N to D
	Agree	13 (31.0)	15 (35.7)	
	Neutral	18 (42.9)	10 (23.8)	
	Disagree	4 (9.5)	11 (26.2)	
	Strongly Disagree	0 (0.0)	0 (0.0)	
**We do not have enough money within our department for tick surveillance**	Strongly Agree	6 (14.3)	4 (9.5)	0.2482 N to A
	Agree	10 (23.8)	12 (28.6)	
	Neutral	20 (47.6)	18 (42.9)	
	Disagree	6 (14.3)	8 (19.0)	
	Strongly Disagree	0 (0.0)	0 (0.0)	
**Tick surveillance is important in Illinois**	Strongly Disagree	0 (0.0)	0 (0.0)	0.2672 N to A
	Disagree	0 (0.0)	0 (0.0)	
	Neutral	4 (9.5)	0 (0.0)	
	Agree	27 (64.3)	29 (69.0)	
	Strongly Agree	11 (26.2)	13 (31.0)	
**Tick surveillance is important in my department’s jurisdiction**	Strongly Disagree	0 (0.0)	0 (0.0)	0.8994 N to A A to StA
	Disagree	2 (4.8)	1 (2.4)	
	Neutral	9 (21.4)	4 (9.5)	
	Agree	25 (59.5)	28 (66.7)	
	Strongly Agree	5 (11.9)	9 (21.4)	
	No Response	1 (2.4)	0 (0.0)	
**I am worried about tick-borne diseases in Illinois**	Strongly Disagree	0 (0.0)	0 (0.0)	0.1237 N to A
	Disagree	3 (7.1)	1 (2.4)	
	Neutral	9 (21.4)	5 (11.9)	
	Agree	25 (59.5)	28 (66.7)	
	Strongly Agree	5 (11.9)	8 (19.0)	
**I do not think tick-borne diseases are a problem in my county/jurisdiction**	Strongly Agree	0 (0.0)	0 (0.0)	0.2888 N to D D to StD
	Agree	2 (4.8)	2 (4.8)	
	Neutral	8 (19.0)	4 (9.5)	
	Disagree	25 (59.5)	25 (59.5)	
	Strongly Disagree	7 (16.7)	11 (26.2)	
**Tick-borne diseases are not a public health problem**	Strongly Agree	0 (0.0)	1 (2.4)	0.4795 N to D
	Agree	1 (2.4)	2 (4.8)	
	Neutral	3 (7.1)	0 (0.0)	
	Disagree	21 (50.0)	23 (54.8)	
	Strongly Disagree	17 (40.5)	16 (38.1)	
**Attitudes Score (Mean (SD))**		39.10 (4.11)	44.38 (4.38)	< 0.0001

In *P*-value column: *StD* strongly disagree, *D* disagree, *N* neutral, *A* agree, *StA* strongly agree

### Practices

Close to 43% (24/56) of post-training participants agreed they would be increasing the amount of tick surveillance they would be performing, as compared to 38% (23/60) prior to training. Before the training, 15% (9/60) of participants planned on performing tick surveillance in 2019. Following the training, this percent increased to almost 20% (11/56). After six months, 29% (5/17) of the follow-up participants reported performing some form of tick surveillance in 2019 (Suppl Table 3). There were no significant differences in the individual questions or combined practice scores between timepoints from the participants who completed both the pre- and post- surveys (Tables 5). Total attitude and practices scores were found to have significant (*p* <  0.01) positive correlation in both pre- (0.402) and post-surveys (0.403).

**Table 5:** Paired responses to practice questions for pre- and post- questionnaires

		**Pre-training (*N* = 42)**	**Post-training** **(*N* = 42)**	
**Question**	**Response**	**Number (%)**	**Number (%)**	**P-value**
**I will be increasing the amount of tick surveillance I perform in the future**	Strongly Disagree	0 (0.0)	0 (0.0)	0.773 N to A
	Disagree	1 (2.4)	0 (0.0)	
	Neutral	24 (57.1)	2 (4.8)	
	Agree	17 (40.5)	18 (42.9)	
	Strongly Agree	0 (0.0)	21 (50.0)	
	No Response	0 (0.0)	1 (2.4)	
**Do you plan to perform any tick surveillance in 2019? (Follow-up: Did you perform surveillance in 2019?)**	No	11 (26.2)	7 (16.7)	0.5566
	Maybe	26 (61.9)	26 (61.9)	
	Yes	5 (11.9)	9 (21.4)	
**Practices Score** **(Median (IQR))**		4 (4–5)	5 (4–5)	0.2065

IQR Interquartile range, In *P*-value column: *N* neutral, *A* agree

## Discussion

Tick and tick-borne disease surveillance are pivotal tools in the prevention and control of TBDs, but, historically, surveillance efforts have suffered from an absence of systematic collection methods [14]. Additionally, federal and state public health agencies often rely on local health departments to perform surveillance even though these local health departments feel they lack the money, guidance, time, and training to perform such surveillance effectively [16]. In this study, we provided in-person tick surveillance training workshops, based on the 2019 CDC guidelines for *I. scapularis* surveillance, to LHD employees within Illinois [17]. Repeated cross-sectional surveys were conducted to evaluate how this training effected the knowledge, attitudes, and practices of these employees regarding tick and tick-borne pathogen surveillance. We recognize that a limitation of the current study-design is that there is a risk that the participants only retain the answers to the questions asked, before the training. However, to limit this risk, we did not allow the participants to have access to the questions in front of them while going through the training and they were not aware the questions would be the same from pre to post. Furthermore, we asked a total of forty-two questions after three hours decreasing the chances to memorize all the questions presented to them on the pre-survey. We also did not provide the answer key to the pre-survey at any point during the training. Whilst there has been a similar study conducted with mosquito control agencies [20], to our knowledge, this is the first such study to look at the impacts of tick surveillance training in LHD employees.

At least one employee from 40 different local health departments received training on tick and tick-borne pathogen surveillance through our study, and 80% of workshop attendees also agreed to take part in at least one KAP survey. While every IDPH Environmental Health Region was represented, the largest proportion of participants were from the northern regions of the state. Increased participation from the northern portions of the state could be related to the geographic distribution of *I. scapularis* within the state. Since first being reported in northern Illinois in the late 80 s, *I. scapularis* has expanded its range to become one of the most predominant tick species in that region [21]. *Ixodes scapularis* is the primary vector of Lyme disease in the eastern half of the US, and its distribution corresponds with the areas of highest Lyme disease incidence within Illinois [3]. The higher rates of Lyme disease and anaplasmosis make tick and tick-borne pathogen surveillance of increased public health importance in this region and may account for increase participation. Unsurprisingly, most of the participants in the training and KAP surveys, no matter the region, worked for the environmental health division of their local heal department. This division is usually in charge of vector surveillance, therefore the most motivated to attend the training.

Overall knowledge scores were low in participants prior to training, with only 16.7% of all baseline participants getting more that 50% of the questions correct. This increased to 96% in post-training and 88% after six months. Participants who completed both pre- and post-training surveys had significant increases in their average knowledge scores. We did not ask follow-up questions about how the knowledge gained during training was used beyond asking if participants had performed actual active tick surveillance. It is possible that other elements outside of our training efforts influenced knowledge retention (i.e., additional training through work, pet ownership, personal experiences with ticks). When looking by subcategory, the training had the largest effect on knowledge of ticks and tick identification, where mean scores increased, on average, by over 2.5 points after training and by 1.5 points after six months. Prior to training there were 2% of participants who answered 70% of the questions correctly in this category, but immediately after training it went up to 80%. Even after six months the percentage was still at 66.7%. While the total baseline knowledge of ticks appeared to be low, ability to correctly identify tick species from a picture was high. In our survey, 58% of baseline participants were able to identify the picture of a Lone star tick (*A. americanum*). According to one study, this is two times more accurate than a member of the public [22]. After training, over 80% of participants were able to identify the tick correctly. This significant increase in knowledge may be attributed to the practical experience provided during the training.

Before receiving training, the participants in this study answered an average of 43% of safety/protective practices questions and 29% of tick-borne disease questions correctly. Although this is similar to knowledge scores in residents of Lyme disease endemic areas [23], we considered this low for our study’s population of public health professionals, especially when thinking about implementing an active tick surveillance program. There is a higher risk of infection with a TBDs when living or working in habitats where densities of infected ticks are high, and increased knowledge on TBDs has been correlated with increased preventive practices [24, 25]. Studies in U.S. Forest Service employees and public health nurses have found knowledge scores related to TBDs or personal protective measures to be in the 80–90%, respectively [26, 27]. Both populations had historically received training or educational materials related to tick safety and/or TBDs. After our training, participants were able to answer 80% of the safety questions and close to 60% of the TBDs questions correctly. While this level of knowledge might not be as high as the previous studies, the differences in means from pre- to post-training were found to be significant, and the effect of the training was found to be high.

The final subcategory within the knowledge sections was related to the actual methods of performing active tick surveillance per the CDC guidelines [17]. Interestingly, this section had some of the lowest and highest number of correct responses per question. For example, the question related to quantifying the densities of host-seeking female ticks only had five (11.9%) correct responses prior to the training. This is not surprising since the guidelines did not come out until early 2019 and this training occurred in April and May of that year. Also, most active tick surveillance by local health departments has primarily been focused on determining the presence or absence of the vector species and not on determining the density of those species or the pathogens they carry [16]. On the other hand, the question in this section about the use of ethanol to preserve tick specimens was one of the most correct (~ 90–100% across all three surveys). This juxtaposition is probably related to the mosquito surveillance and control activities participants perform as part their jobs in environmental health. Local health departments perform 42% of the vector control services in the U.S. and prioritized mosquito monitoring within those services [28]. Because of this, the participants likely already had a basic understanding that can be applied across a variety of vector species. While a significant increase in knowledge was seen immediately after training, the specificity of some of the methods questions would have made answering correctly difficult if prior knowledge had not been applied.

Following training, there were significant increases in the average overall attitude score as well as the median scores for perceived need, perceived barriers, and perceived risks of TBDs. These increases coincide with increased positive attitudes towards performing tick surveillance. At least 80% of participants believed that TBDs were a public health problem, and over 70% indicated it was a problem in their own jurisdiction. High perceived risk of tick-borne diseases, such as that observed in our study, has been associated with increased willingness to perform preventive practices [27, 29, 30]. This was further confirmed by the significant positive correlation between perceived risk and perceived need for surveillance in our study following training.

The Health Belief Model is a framework that has been commonly used to study what makes individuals adopt preventive behaviors related to TBDs [24, 29, 31]. Perceived barriers, one component of the Health Belief Model, has been found to be one of the most important determinants in adopting preventive practices [32]. Prior to the training, more participants agreed that surveillance was limited by a lack of knowledge, rather than a lack of time or money. This completely reversed following the training. In fact, the only significant comparison within individual questions was related to the ability to identify the main vector tick species in Illinois. Only 20% of respondent initial believed they could identify the ticks, but after training it increased to over 50%. We believe these changes in attitude are related to the practical experience provided during the training. A study that offered tick surveillance training to a very similar population reported participants believed a more hands on experiences with tick identification would have improved the training experience [20]. The other knowledge as barriers questions saw similar trends, leading to perceived barriers being the only category with a significant increase (here an increase is a reduction in barriers) in median from pre- to post-training.

Increases in positive attitudes towards tick surveillance were associated with increased intentions to perform surveillance prior to and immediately after training. The proportion of participants planning to perform tick surveillance did increase from 15 to 20%, which was not statistically significant. There are conflicting reports on the effectiveness of increasing knowledge to promote preventative practices, but in the case of this study a significant association was not observed [27, 30, 33, 34]. The lack of significant changes in practices may be related to some of the limitations of our study. We were using a convenience sample that relied on voluntary participation of LHD employees, a population that is notoriously overworked. This led to both a small sample size and a retention rate of only 20% for the third and final survey. For this reason, more in-depth statistical comparisons between the pre- or post- surveys and the follow-up survey were not included in our analysis. In addition, we did not collect demographic information that might be used to help explain the lack of significant findings. Beyond these limitations, the findings could be related to the focus of our study. Almost all of the KAP studies on tick-borne diseases are focused on the individual level instead of the organizational level because the individual level is where the emphasis for TBDs preventive practices and control has historically been focused [35]. Now, with the expanding risk of TBDs, the focus has started to shift towards developing integrated tick-management programs. To go along with this, the target populations of KAP surveys may need to be geared toward employees of organizations that will be implementing community wide preventions related to these programs as we have done here.

## Conclusion

With the rising number of tick-borne disease case in the U.S., the geographic expansion of tick species, and the continued discovery of new pathogens and invasive tick species, tick surveillance is needed now more than ever. Local health departments are a key ingredient in implementing surveillance as a public health tool, but they often lack the support and training needed to perform effectively. Our study addressed one of these barriers by providing and evaluating training within LHD employees. At a time when there was no active surveillance of ticks and TBD in Illinois, the training was proven to be effective in increasing the knowledge of ticks, tick-borne diseases, and surveillance as well as promoting positive attitudes related to surveillance. Probably the most appreciated and effective aspect of the training was providing practical experience with tick identification. While the training, by itself, was not associated with increases in surveillance practices, we were able to empower local public health officials with the knowledge and positive attitudes needed to enact change. They can, in turn, impart those characteristics to the populations they serve. Future studies may want to consider more hands-on teaching methods that focus on finding ways to reduce other barriers, such as lack of funding and time, within this population.

## Supplementary Information

**Additional file 1. **

## Data Availability

The questionnaires and datasets used during the current study are available upon request to corresponding author, or at (https://databank.illinois.edu/datasets/IDB-6268941?code=5VF-vFTzImWBuJiGePeKBnSz_uCLE4Vyp1_EoBXYpcw).

## Abbreviations

TBD: Tick-borne disease
CDC: Centers for Disease Control and Prevention
LHD: Local health department
IDPH: Illinois Department of Public Health
KAP: Knowledge Attitudes and Practices survey
M: Mean
SD: Standard deviation
Mdn: Median
IQR: Interquartile range
StD: Strongly disagree
D: Disagree
N: Neutral
A: Agree
StA: Strongly agree
US: United States
