# Supplementary material for: Effects of tick surveillance education on knowledge, attitudes, and practices of local health department employees
Source: BMC Public Health. 2022 Feb 2;22:215. doi: 10.1186/s12889-022-12667-2 (PMC8812035; doi:10.1186/s12889-022-12667-2)
Supplement: Supplementary file 1 — Additional file 1. [file 12889_2022_12667_MOESM1_ESM.docx]

**Supplemental Tables**

Supplemental Table 1: Knowledge questions with responses from all respondents

|  |  | **Pre-training (N=60)** | **Post-training (N=56)** | **Six-months follow-up  (N=17)** |
| --- | --- | --- | --- | --- |
| **Question** | **Response** | **Number (%)** | **Number (%)** | **Number (%)** |
| **Which tick life stages have 8 legs?** | Incorrect | 40 (66.7) | 10 (17.9) | 7 (41.2) |
|  | Correct | 20 (33.3) | 46 (82.1) | 10 (58.8) |
| **The "head" of a tick is called the hypostome and is made up of the palps, the chelicerae, and the capitulum.** | Incorrect | 49 (81.7) | 35 (62.5) | 13 (76.5) |
|  | Correct | 11 (18.3) | 21 (37.5) | 4 (23.5) |
| **What sex is the tick in the picture below?** | Incorrect | 22 (36.7) | 1 (1.8) | 4 (23.5) |
|  | Correct | 38 (63.3) | 55 (98.2) | 13 (76.5) |
| **What tick species is pictured below?** | Incorrect | 25 (41.7) | 10 (17.9) | 0 (0.0) |
|  | Correct | 35 (58.3) | 46 (82.1) | 17 (100.0) |
| **Which species of tick has an anal groove seen in the picture below?** | Incorrect | 51 (85.0) | 16 (28.6) | 7 (41.2) |
|  | Correct | 9 (15.0) | 40 (71.4) | 10 (58.8) |
| **What are the ridges called that run along the bottom abdominal border of some tick species (see picture below)?** | Incorrect | 48 (80.0) | 1 (1.8) | 2 (11.8) |
|  | Correct | 12 (20.0) | 55 (98.2) | 15 (88.2) |
| **Which of the following is an insecticide?** | Incorrect | 27 (45.0) | 4 (7.1) | 2 (11.8) |
|  | Correct | 33 (55.0) | 52 (92.9) | 15 (88.2) |
| **All of the following are false when removing an attached tick except?** | Incorrect | 43 (71.7) | 22 (39.3) | 7 (41.2) |
|  | Correct | 17 (28.3) | 34 (60.7) | 10 (58.8) |
| **Which of the following are common places to find ticks on your body?** | Incorrect | 33 (55.0) | 5 (8.9) | 5 (29.4) |
|  | Correct | 27 (45.0) | 51 (91.1) | 12 (70.6) |
| **After treating clothing with permethrin, you must allow it to dry for at least 12 hours before wearing.** | Incorrect | 36 (60.0) | 13 (23.2) | 10 (58.8) |
|  | Correct | 24 (40.0) | 43 (76.8) | 7 (41.2) |
| **There are many other safety concerns involved with tick surveillance besides tick bites.** | Incorrect | 6 (10.0) | 2 (3.6) | 1 (5.9) |
|  | Correct | 54 (90.0) | 54 (96.4) | 16 (94.1) |
| **If performing tick surveillance during hunting season, which of the following is false?** | Incorrect | 38 (63.3) | 11 (19.6) | 6 (35.3) |
|  | Correct | 22 (36.7) | 45 (80.4) | 11 (64.7) |
| **The four most common tick-borne disease in Illinois are Lyme disease, Anaplasmosis, Tularemia, and Ehrlichiosis.** | Incorrect | 47 (78.3) | 38 (67.9) | 13 (76.5) |
|  | Correct | 13 (21.7) | 18 (32.1) | 4 (23.5) |
| **Which of the following are true about tick-borne diseases?** | Incorrect | 53 (88.3) | 52 (92.9) | 13 (76.5) |
|  | Correct | 7 (11.7) | 4 (7.1) | 4 (23.5) |

Supplemental Table 1: (cont.)

|  |  | **Pre-training (N=60)** | **Post-training (N=56)** | **Six-months follow-up  (N=17)** |
| --- | --- | --- | --- | --- |
| **Question** | **Response** | **Number (%)** | **Number (%)** | **Number (%)** |
| **Which of the following is not a tick-borne disease?** | Incorrect | 41 (68.3) | 4 (7.1) | 4 (23.5) |
|  | Correct | 19 (31.7) | 52 (92.9) | 13 (76.5) |
| **Erythema migrans ("bullseye" rash) is only seen with Lyme disease (see picture below).** | Incorrect | 39 (65.0) | 19 (33.9) | 7 (41.2) |
|  | Correct | 21 (35.0) | 37 (66.1) | 10 (58.8) |
| **Which pathogen causes Rocky Mountain Spotted Fever?** | Incorrect | 23 (38.3) | 8 (14.3) | 4 (23.5) |
|  | Correct | 37 (61.7) | 48 (85.7) | 13 (76.5) |
| **Within Illinois, which tick species is most likely to transmit Anaplasmosis?** | Incorrect | 43 (71.7) | 21 (37.5) | 3 (17.6) |
|  | Correct | 17 (28.3) | 35 (62.5) | 14 (82.4) |
| **How many hosts do the vector ticks of concern in Illinois require to complete their lifecycle?** | Incorrect | 47 (78.3) | 17 (30.4) | 8 (47.1) |
|  | Correct | 13 (21.7) | 39 (69.6) | 9 (52.9) |
| **Which tick species will you encounter more frequently in grassy fields?** | Incorrect | 32 (53.3) | 20 (35.7) | 5 (29.4) |
|  | Correct | 28 (46.7) | 36 (64.3) | 12 (70.6) |
| **When is a tick species considered to be established within a county?** | Incorrect | 30 (50.0) | 15 (26.8) | 9 (52.9) |
|  | Correct | 30 (50.0) | 41 (73.2) | 8 (47.1) |
| **Which of the following are acceptable tick collection methods for classifying the county status of tick species?** | Incorrect | 47 (78.3) | 16 (28.6) | 15 (88.2) |
|  | Correct | 13 (21.7) | 40 (71.4) | 2 (11.8) |
| **How often should drags be inspected for ticks (according to CDC guidelines) when you want to calculate the density of host-seeking nymphs?** | Incorrect | 34 (56.7) | 23 (41.1) | 8 (47.1) |
|  | Correct | 26 (43.3) | 33 (58.9) | 9 (52.9) |
| **How many sites must be sampled per county (according to CDC guidelines) when you want to calculate density of host-seeking females?** | Incorrect | 52 (86.7) | 37 (66.1) | 12 (70.6) |
|  | Correct | 8 (13.3) | 19 (33.9) | 5 (29.4) |
| **Ticks can be preserved in 70-95% ethanol.** | Incorrect | 7 (11.7) | 3 (5.4) | 1 (5.9) |
|  | Correct | 53 (88.3) | 53 (94.6) | 16 (94.1) |
| **The "fingers" or "strips" on a drag along with weights sewn into the trailing edge are there to increase contact between the fabric and vegetation.** | Incorrect | 4 (6.7) | 0 (0.0) | 0 (0.0) |
|  | Correct | 56 (93.3) | 56 (100.0) | 17 (100.0) |
| **Knowledge Score  (Mean (SD))** |  | 10.72 (3.15) | 18.80 (2.93) | 16.24 (2.99) |

Supplemental Table 2: All Participant Responses to Attitudes Questions

|  |  | **Pre-training (N=60)** | **Post-training (N=56)** | **Six-months follow-up  (N=17)** |
| --- | --- | --- | --- | --- |
| **Question** | **Response** | **Number (%)** | **Number (%)** | **Number (%)** |
| **Tick surveillance is needed within my jurisdiction.** | Strongly Disagree | 0 (0.0) | 0 (0.0) | 0 (0.0) |
|  | Disagree | 1 (1.7) | 1 (1.8) | 0 (0.0) |
|  | Neutral | 7 (11.7) | 2 (3.6) | 2 (11.8) |
|  | Agree | 35 (58.3) | 35 (62.5) | 10 (58.8) |
|  | Strongly Agree | 10 (16.7) | 10 (17.9) | 5 (29.4) |
|  | No Response | 7 (11.7) | 8 (14.3) | 0 (0.0) |
| **My department is already doing enough tick surveillance.** | Strongly Agree | 0 (0.0) | 1 (1.8) | 0 (0.0) |
|  | Agree | 4 (6.7) | 3 (5.4) | 2 (11.8) |
|  | Neutral | 12 (20.0) | 11 (19.6) | 5 (29.4) |
|  | Disagree | 28 (46.7) | 27 (48.2) | 10 (58.8) |
|  | Strongly Disagree | 9 (15.0) | 6 (10.7) | 0 (0.0) |
|  | No Response | 7 (11.7) | 8 (14.3) | 0 (0.0) |
| **I do not feel like I have enough knowledge and preparation to do tick surveillance accurately.** | Strongly Agree | 13 (21.7) | 1 (1.8) | 0 (0.0) |
|  | Agree | 26 (43.3) | 9 (16.1) | 5 (29.4) |
|  | Neutral | 4 (6.7) | 14 (25.0) | 5 (29.4) |
|  | Disagree | 9 (15.0) | 23 (41.1) | 7 (41.2) |
|  | Strongly Disagree | 0 (0.0) | 1 (1.8) | 0 (0.0) |
|  | No Response | 8 (13.3) | 8 (14.3) | 1 (5.9) |
| **I do not feel like I have enough knowledge and preparation to do tick surveillance safely.** | Strongly Agree | 12 (20.0) | 0 (0.0) | 0 (0.0) |
|  | Agree | 18 (30.0) | 6 (10.7) | 4 (23.5) |
|  | Neutral | 11 (18.3) | 9 (16.1) | 2 (11.8) |
|  | Disagree | 11 (18.3) | 30 (53.6) | 8 (47.1) |
|  | Strongly Disagree | 0 (0.0) | 3 (5.4) | 2 (11.8) |
|  | No Response | 8 (13.3) | 8 (14.3) | 0 (0.0) |
| **I feel confident I can identify the four main vector tick species within IL.** | Strongly Disagree | 12 (20.0) | 0 (0.0) | 0 (0.0) |
|  | Disagree | 21 (35.0) | 5 (8.9) | 6 (35.3) |
|  | Neutral | 6 (10.0) | 12 (21.4) | 4 (23.5) |
|  | Agree | 9 (15.0) | 29 (51.8) | 7 (41.2) |
|  | Strongly Agree | 3 (5.0) | 2 (3.6) | 0 (0.0) |
|  | No Response | 9 (15.0) | 8 (14.3) | 0 (0.0) |

Supplemental Table 2: (cont.)

|  |  | **Pre-training (N=60)** | **Post-training (N=56)** | **Six-months follow-up  (N=17)** |
| --- | --- | --- | --- | --- |
| **Question** | **Response** | **Number (%)** | **Number (%)** | **Number (%)** |
| **I do not feel like I have enough time for tick surveillance within my job.** | Strongly Agree | 7 (11.7) | 7 (12.5) | 4 (23.5) |
|  | Agree | 17 (28.3) | 19 (33.9) | 6 (35.3) |
|  | Neutral | 22 (36.7) | 11 (19.6) | 4 (23.5) |
|  | Disagree | 6 (10.0) | 11 (19.6) | 2 (11.8) |
|  | Strongly Disagree | 0 (0.0) | 0 (0.0) | 1 (5.9) |
|  | No Response | 8 (13.3) | 8 (14.3) | 0 (0.0) |
| **We do not have enough money within our department for tick surveillance.** | Strongly Agree | 7 (11.7) | 4 (7.1) | 4 (23.5) |
|  | Agree | 15 (25.0) | 17 (30.4) | 5 (29.4) |
|  | Neutral | 22 (36.7) | 19 (33.9) | 5 (29.4) |
|  | Disagree | 8 (13.3) | 8 (14.3) | 3 (17.6) |
|  | Strongly Disagree | 0 (0.0) | 0 (0.0) | 0 (0.0) |
|  | No Response | 8 (13.3) | 8 (14.3) | 0 (0.0) |
| **Tick surveillance is important in Illinois.** | Strongly Disagree | 0 (0.0) | 0 (0.0) | 0 (0.0) |
|  | Disagree | 0 (0.0) | 0 (0.0) | 0 (0.0) |
|  | Neutral | 4 (6.7) | 0 (0.0) | 1 (5.9) |
|  | Agree | 29 (48.3) | 34 (59.6) | 8 (47.1) |
|  | Strongly Agree | 19 (31.7) | 15 (26.3) | 8 (47.1) |
|  | No Response | 8 (13.3) | 8 (14.3) | 0 (0.0) |
| **Tick surveillance is important in my department’s jurisdiction.** | Strongly Disagree | 0 (0.0) | 0 (0.0) | 0 (0.0) |
|  | Disagree | 2 (3.3) | 1 (1.8) | 1 (5.9) |
|  | Neutral | 10 (16.7) | 7 (12.5) | 4 (23.5) |
|  | Agree | 31 (51.7) | 31 (55.4) | 6 (35.3) |
|  | Strongly Agree | 8 (13.3) | 9 (16.1) | 6 (35.3) |
|  | No Response | 9 (15.0) | 8 (14.3) | 0 (0.0) |
| **I am worried about tick-borne diseases in Illinois.** | Strongly Disagree | 0 (0.0) | 0 (0.0) | 0 (0.0) |
|  | Disagree | 3 (5.0) | 1 (1.8) | 1 (5.9) |
|  | Neutral | 10 (16.7) | 6 (10.7) | 5 (29.4) |
|  | Agree | 30 (50.0) | 32 (57.1) | 7 (41.2) |
|  | Strongly Agree | 9 (15.0) | 9 (16.1) | 4 (23.5) |
|  | No Response | 8 (13.3) | 8 (14.3) | 0 (0.0) |
| **I do not think tick-borne diseases are a problem in my county/jurisdiction.** | Strongly Agree | 0 (0.0) | 0 (0.0) | 0 (0.0) |
|  | Agree | 2 (3.3) | 2 (3.6) | 2 (11.8) |
|  | Neutral | 8 (13.3) | 5 (8.9) | 1 (5.9) |
|  | Disagree | 29 (48.3) | 29 (51.8) | 10 (58.8) |
|  | Strongly Disagree | 13 (21.7) | 12 (21.4) | 4 (23.5) |
|  | No Response | 8 (13.3) | 8 (14.3) | 0 (0.0) |

Supplemental Table 2: (cont.)

|  |  | **Pre-training (N=60)** | **Post-training (N=56)** | **Six-months follow-up  (N=17)** |
| --- | --- | --- | --- | --- |
| **Question** | **Response** | **Number (%)** | **Number (%)** | **Number (%)** |
| **Tick-borne diseases are not a public health problem.** | Strongly Agree | 0 (0.0) | 1 (1.8) | 0 (0.0) |
|  | Agree | 1 (1.7) | 2 (3.6) | 0 (0.0) |
|  | Neutral | 3 (5.0) | 0 (0.0) | 0 (0.0) |
|  | Disagree | 24 (40.0) | 26 (46.4) | 10 (58.8) |
|  | Strongly Disagree | 24 (40.0) | 19 (33.9) | 7 (41.2) |
|  | No Response | 8 (13.3) | 8 (14.3) | 0 (0.0) |
| **Attitude Score (Mean (SD))** |  | 39.60 (6.44) | 44.17 (4.17) | 42.53 (5.58) |

Supplemental Table 3: Participant Responses to Practices Questions

|  |  | **Pre-training (N=60)** | **Post-training (N=56)** | **Six-months follow-up  (N=17)** |
| --- | --- | --- | --- | --- |
| **Question** | **Response** | **Number (%)** | **Number (%)** | **Number (%)** |
| **I will be increasing the amount of tick surveillance I perform in the future.** | Strongly Disagree | 0 (0.0) | 0 (0.0) | 0 (0.0) |
|  | Disagree | 1 (1.7) | 3 (5.4) | 2 (11.8) |
|  | Neutral | 28 (46.7) | 20 (35.7) | 12 (70.6) |
|  | Agree | 21 (35.0) | 24 (42.9) | 3 (17.6) |
|  | Strongly Agree | 2 (3.3) | 0 (0.0) | 0 (0.0) |
|  | No Response | 8 (13.3) | 9 (16.1) | 0 (0.0) |
| **Do you plan to perform any tick surveillance in 2019? (Follow-up: Did you perform surveillance in 2019?)** | No | 19 (31.7) | 14 (25.0) | 12 (70.6) |
|  | Maybe | 32 (53.3) | 31 (55.4) | 0 (0.0) |
|  | Yes | 9 (15.0) | 11 (19.6) | 5 (29.4) |
| **Practices Score**  **(Median (IQR))** |  | 4 (3-5) | 4 (3-5) | 3 (3-5) |
